# Comparison of plasma and neuroimaging biomarkers to predict cognitive decline in non-demented memory clinic patients

**DOI:** 10.1186/s13195-024-01478-9

**Published:** 2024-05-16

**Authors:** Augusto J. Mendes, Federica Ribaldi, Aurelien Lathuiliere, Nicholas J. Ashton, Henrik Zetterberg, Marc Abramowicz, Max Scheffler, Frédéric Assal, Valentina Garibotto, Kaj Blennow, Giovanni B. Frisoni

**Affiliations:** 1 Laboratory of Neuroimaging of Aging (LANVIE), University of Geneva, Geneva, Switzerland; 2 Geneva Memory Center, Department of Rehabilitation and Geriatrics,, Geneva University Hospitals, Geneva, Switzerland; 3 Department of Psychiatry and Neurochemistry, Institute of Neuroscience & Physiology, The Sahlgrenska Academy at the University of Gothenburg, Mölndal, Sweden; 4 King’s College London, Institute of Psychiatry, Psychology and Neuroscience, Maurice Wohl Clinical Neuroscience Institute, London, UK; 5 NIHR Biomedical Research Centre for Mental Health and Biomedical Research Unit for Dementia at South London and Maudsley NHS Foundation, London, UK; 6 Centre for Age-Related Medicine, Stavanger University Hospital, Stavanger, Norway; 7 Clinical Neurochemistry Laboratory, Sahlgrenska University Hospital, Mölndal, Sweden; 8 Department of Neurodegenerative Disease, UCL Institute of Neurology, Queen Square, London UK; 9 UK Dementia Research Institute at UCL, London, UK; 10 Hong Kong Center for Neurodegenerative Diseases, Clear Water Bay, Hong Kong, China; 11 Wisconsin Alzheimer?s Disease Research Center, University of Wisconsin School of Medicine and Public Health, Madison, WI USA; 12 Genetic Medicine, Diagnostics Dept, University Hospitals and University of Geneva, Geneva, Switzerland; 13 Division of Radiology, Geneva University Hospitals, Geneva, Switzerland; 14 Division of Neurology, Department of Clinical Neurosciences, Faculty of Medicine, Geneva University Hospitals, University of Geneva, Geneva, Switzerland; 15 Laboratory of Neuroimaging and Innovative Molecular Tracers (NIMTlab), Faculty of Medicine, Geneva University Neurocenter, University of Geneva, Geneva, Switzerland; 16 Division of Nuclear Medicine and Molecular Imaging, Geneva University Hospitals, Geneva, Switzerland; 17 CIBM Center for Biomedical Imaging, Geneva, Switzerland; 18 Department of Psychiatry and Neurochemistry, University of Gothenburg, Gothenburg, Sweden

## Abstract

**Background:**

Plasma biomarkers of Alzheimer’s disease (AD) pathology, neurodegeneration, and neuroinflammation are ideally suited for secondary prevention programs in self-sufficient persons at-risk of dementia. Plasma biomarkers have been shown to be highly correlated with traditional imaging biomarkers. However, their comparative predictive value versus traditional AD biomarkers is still unclear in cognitively unimpaired (CU) subjects and with mild cognitive impairment (MCI).

**Methods:**

Plasma (Aβ42/40, p-tau181, p-tau231, NfL, and GFAP) and neuroimaging (hippocampal volume, centiloid of amyloid-PET, and tau-SUVR of tau-PET) biomarkers were assessed at baseline in 218 non-demented subjects (CU = 140; MCI = 78) from the Geneva Memory Center. Global cognition (MMSE) was evaluated at baseline and at follow-ups up to 5.7 years. We used linear mixed-effects models and Cox proportional-hazards regression to assess the association between biomarkers and cognitive decline. Lastly, sample size calculations using the linear mixed-effects models were performed on subjects positive for amyloid-PET combined with tau-PET and plasma biomarker positivity.

**Results:**

Cognitive decline was significantly predicted in MCI by baseline plasma NfL (β=-0.55), GFAP (β=-0.36), hippocampal volume (β = 0.44), centiloid (β=-0.38), and tau-SUVR (β=-0.66) (all *p* < 0.05). Subgroup analysis with amyloid-positive MCI participants also showed that only NfL and GFAP were the only significant predictors of cognitive decline among plasma biomarkers. Overall, NfL and tau-SUVR showed the highest prognostic values (hazard ratios of 7.3 and 5.9). Lastly, we demonstrated that adding NfL to the inclusion criteria could reduce the sample sizes of future AD clinical trials by up to one-fourth in subjects with amyloid-PET positivity or by half in subjects with amyloid-PET and tau-PET positivity.

**Conclusions:**

Plasma NfL and GFAP predict cognitive decline in a similar manner to traditional imaging techniques in amyloid-positive MCI patients. Hence, even though they are non-specific biomarkers of AD, both can be implemented in memory clinic workups as important prognostic biomarkers. Likewise, future clinical trials might employ plasma biomarkers as additional inclusion criteria to stratify patients at higher risk of cognitive decline to reduce sample sizes and enhance effectiveness.

**Supplementary Information:**

The online version contains supplementary material available at 10.1186/s13195-024-01478-9.

## Introduction

Alzheimer’s disease (AD) pathology is defined by the accumulation of amyloid plaques (A), tau neurofibrillary tangles (T), and neurodegeneration (N) [1]. These biomarkers can be assessed using magnetic resonance imaging (MRI), positron emission tomography (PET), or cerebrospinal fluid (CSF). However, all of these are either expensive or invasive techniques even though they are accurate in measuring the AD biomarkers. Recently, plasma biomarkers proved to be a promising tool to identify AD pathology and track disease progression, finding of great importance due to their accessibility, affordability, and acceptability. For instance, plasma Aβ42/40, phosphorylated tau at threonine 181 (p-tau181), 231 (p-tau231), and neurofilament light chain (NfL) are associated respectively with ATN model measures [2–4]. Thus, implementing plasma biomarkers in the diagnostic workup could spare a significant number of expensive traditional exams to patients and improve the cost effectiveness of health services [5].

Traditional neuroimaging biomarkers have been shown to identify non-demented subjects at high risk for cognitive decline. The amyloid load evaluated by PET or CSF can predict cognitive decline in cognitively unimpaired (CU) subjects [6] and subjects with mild cognitive impairment (MCI) [7]. Likewise, hippocampal volume also significantly predicted the progression to dementia in MCI subjects [8]. Nonetheless, when compared to amyloid and hippocampal volume, tau PET was the strongest predictor of cognitive decline in the AD continuum, from CU to AD [9, 10].

In line with the evidence from neuroimaging, plasma biomarkers were found to be able to predict cognitive decline in CU and MCI. Baseline plasma Aβ42/40 and NfL concentrations were able to predict cognitive decline in a CU research population [11]. Likewise, Cullen and colleagues [12] found similar results for plasma Aβ42/40, NfL, and p-tau217. P-tau181 was also found to be efficient in predicting cognitive changes and grey matter changes in MCI and CU subjects, whereas NfL predicted cognitive deterioration only in cognitively impaired subjects [4, 13]. Further, p-tau231, a plasma marker which increases early with Aβ dysmetabolism [14], has been shown to predict increases in Aβ PET signal over 3 years [14]. In MCI subjects, baseline p-tau181 and NfL combined showed the best predictor model regarding cognitive decline, whilst Aβ42/40 was not predictive [15]. A *post-mortem* study showed similar findings, namely that p-tau181, p-tau231, and NfL levels were significant predictors of a steeper cognitive decline, while no significant result was found for Aβ42/40 [16]. Another study revealed similar effects for levels of p-tau181, NfL, and glial fibrillary acidic protein (GFAP); however, the Aβ42/40 was once again not a significant predictor of cognitive deterioration [17].

Overall, recent evidence suggests the potential of plasma biomarkers in the identification of at-risk subjects. However, the utility of these biomarkers in predicting cognitive changes might vary according to several factors including the cognitive status. For instance, baseline Aβ42/40 is useful for identifying at-risk CU subjects, although this is not so evident in MCI [13]. Furthermore, p-tau231 and GFAP also demonstrated their utility, even though evidence is scarce.

However, despite the literature dealing with neuroimaging and plasma biomarkers predicting cognitive changes over time, both biomarkers were mostly tested independently, and little is known about the relative predictive power when comparing them directly. Therefore, this study is intended to: (i) test the association between baseline plasma and neuroimaging biomarkers from the ATN model with cognitive decline in non-demented individuals; (ii) measure the prognostic value of plasma and neuroimaging biomarkers in cognitive decline, and (iii) calculate how adding plasma biomarkers to inclusion criteria could decrease the sample sizes in preventive AD clinical trials.

## Methods

### Participants

The study selected subjects from the Geneva Memory Center (GMC) cohort. All patients underwent diagnostic workup including clinical and neuropsychological evaluations, as well as biomarker assessment either as part of the diagnostic workup itself or in the context of clinical research projects [18]. Non-demented participants with plasma collection within 1.5 years from the last global cognition test and at least one follow-up exam at the GMC were enrolled. For the CU group, we considered all subjects without any cognitive impairment, including worried well, and subjective cognitive decline, whereas MCI participants were included based on objective clinical and cognitive diagnostic criteria [19]. All participants signed an informed consent form prior to enrollment in the study. The Geneva Ethics Committee approved the study (PB_2016 − 01346 and 2020_00403).

### Biomarkers collection, analyses, and assessment

#### Plasma biomarkers

Plasma was collected in EDTA tubes, kept for two hours at room temperature prior to centrifugation (1700 g, 15 min), aliquoted as 500uL in 1.2mL polypropylene tubes, and maintained at -80 °C in the local biobank of the Memory Center of Geneva University Hospitals. Aliquots were sent under secure conditions to the Clinical Neurochemistry Laboratory, University of Gothenburg (Sweden), where they were analyzed. The concentrations of plasma Aβ42, Aβ40, GFAP, and NfL were determined on an HD-X Automated Immunoassay Analyzer using commercially available Single molecule array (Simoa) Assay Kits in accordance with the recommendations from the manufacturer (Quanterix, Billerica, MA; https://www.quanterix.com/simoa-technology/). Alternately, the levels of p-tau181 [20] and p-tau231 [4] were measured using homebrew Simoa assays developed at the Clinical Neurochemistry Laboratory, University of Gothenburg (Sweden).

#### Neuroimaging biomarkers

The amyloid-PET images were acquired using 18 F-florbetapir or 18 F-flutemetamol tracers, while tau-PET images were acquired using 18 F-Flortaucipir using a protocol previously described in detail here [21]. Briefly, 18 F-florbetapir images were acquired 50 min after injection of 200 MBq during 15 min; 18 F-flutemetamol images were acquired 90 min after injection of 150 MBq during 20 min; and 18 F-flortaucipir images were acquired 75 min after injection of 180 MBq during 30 min. Acquisitions were obtained on Siemens Biograph and Biograph Vision scanners (Siemens, Washington, DC), reconstructed using a 3D OSEM iterative reconstruction, corrected for randoms, dead time, normalization, scatter, attenuation, and sensitivity [21]. An in-house pipeline based on SPM12 (Wellcome Department of Cognitive Neurology, London, UK) was used for the PET images processing [21]. Considering that we used two different amyloid-PET tracers, SUVR was converted to the centiloid scale following guidelines from the Global Alzheimer’s Association Interactive Network (GAAIN) [22]. The tau-PET global tau standardized uptake value ratio (SUVR) was computed as the average across parahippocampal gyrus, amygdala, mid-occipital cortex, and inferior temporal cortex [23].

The hippocampal volume was extracted from structural 3T MRI images. The left and right hippocampal volumes were averaged and normalized according to the total intracranial volume. The extraction was performed in FreeSurfer (version7.0–recon-all; https://surfer.nmr.mgh.harvard.edu).

### ATN-C measures

Plasma and neuroimaging biomarkers were included as surrogates of the ATN model. Amyloid measures were plasma Aβ42/40 and the centiloid (A), tau was evaluated with plasma p-tau181 and p-tau231 and the global SUVR (T), while neurodegeneration was assessed by NfL in plasma and the hippocampal volume (N). In addition, plasma GFAP refers to inflammatory processes (I). Our main outcome was the global score of Mini-Mental State Examination (MMSE) at baseline and consequent follow-ups (C).

### Statistical analysis

The differences of baseline demographics, clinical, cognitive, and biomarkers between CU and MCI cohorts were evaluated by Mann-Whitney test for continuous variables and a Chi-squared test for categorical variables. Moreover, to evaluate if the plasma biomarkers were affected by co-morbidities [24], the medical history of cardiovascular diseases, hypertension, hypercholesterolemia, and diabetes was considered. For that, the differences in each plasma biomarker between the groups with and without co-morbidity were performed independently for the CU and MCI groups. This analysis was performed using the Wilcoxon rank sum test, while the correlation between creatinine and plasma biomarkers was assessed using Pearson’s correlation.

Our first aim to test how baseline plasma and neuroimaging biomarkers are associated with cognitive decline over time was tested through multiple linear mixed-effects (LME) models. The MMSE score was the dependent variable, whereas plasma and neuroimaging biomarkers were independent variables for CU and MCI separately. Univariate and multivariate models, corrected by age, sex, and education, were run using each biomarker alone and altogether, respectively. The models had the following structure: MMSE ~ age + sex + education + time * biomarker. An additional multivariate model was performed only with the significant predictors of cognitive decline detected in the total multivariate LME model. The model fit was evaluated using the Akaike information criterion (AIC) and all the models comprised a random intercept and a random slope of time for each subject. In addition, a subgroup analysis was performed on amyloid-PET positive (Amy+) MCI and CU subjects independently based on the visual assessment by an expert in nuclear medicine. In order to ensure a standardized comparison of all biomarkers, the levels of plasma biomarkers were transformed on the logarithmic scale prior to calculation of the z-score, whereas neuroimaging biomarkers were only z-scored (sensitivity analysis with raw values in Table S1 in Supplementary Materials).

The prognostic value of the groups positive to each plasma and neuroimaging biomarker in cognitive decline was evaluated using Cox proportional-hazards (CPH) regression models. Thus, subjects with values greater than the 90% percentile of the distribution in the CU subjects formed the positive group for each biomarker. On the other hand, cognitive decline was dichotomized in each subject if the observed decline was higher than the expected decline found by Schneider and colleagues [25] based on the follow-up duration and subject’s age. In this manner, we categorized each participant as either a “decliner” or “stable” based on the anticipated changes observed in MMSE. The same method was already implemented in a recent study investigating the predictive effect of tau and hypometabolism in cognitive decline in the same cohort [26]. Kaplan-Meier survival curves were plotted to estimate the probability of “cognitive decline” occurring over time different groups, categorized by the presence or absence of each biomarker.

Lastly, a sample size calculation was conducted to assess the potential reduction in sample sizes for future AD clinical trials by including plasma biomarker positivity as part of the inclusion criteria. Therefore, in line with the most recent clinical trials [27, 28], we calculated sample sizes considering Amy + MCI subjects combined with tau-PET and plasma biomarkers positivity identified in the CPH regression models. The number of subjects per arm was calculated aiming for the detection of cognitive decline slowing between 20% and 50% [29]. The power analysis only comprised plasma biomarkers that predicted significant cognitive decline in LME models, and it was based on previous research for mixed models [30]. For that, we considered the random slopes, inter-subject variability, and the residual error of variance from LME models that only included participants that were positive for each combination of positive biomarkers. The calculation considered a clinical trial with a duration of two years and annual assessments with a statistical power of 90% and an alpha level of 5%. The formula for calculating the sample size can be found in the Supplementary Materials [30]. At last, the number of participants to screen for preventive AD clinical trials was estimated considering the positivity rate for each biomarker and the sample size previously calculated. All analyses were performed in R (Version4.2.0).

## Results

### Participants

A total of 218 participants (57% females, mean age = 67.7 [SD = 8.6]) had plasma and cognitive evaluations divided by cognitive stage, namely 140 CU and 78 MCI. However, the subsample with baseline neuroimaging biomarkers was slightly lower, namely a total of 146 (CU = 75, MCI = 71) for hippocampal volume, 105 (CU = 36, MCI = 69) for amyloid-PET, and 84 (CU = 26, MCI = 58) for tau-PET. The average time (in months) between the plasma collection and the baseline MMSE evaluation was 4.3 ± 3.7 for CU and 3.6 ± 3.7 for MCI subjects. Additionally, the baseline plasma and neuroimaging biomarkers were separated by 0.6 ± 5.5 (CU = 0.05 ± 5.4, MCI = 1.4 ± 5.5) for structural MRI, 3.1 ± 6.3 (CU = 5.9 ± 5, MCI = 1.7 ± 6.5) for amy-PET, and 4.4 ± 7.1 (CU = 6.4 ± 5.3, MCI = 3.5 ± 7.6) for tau-PET. The average interval of cognitive evaluation follow-up in our cohort was 30.5 ± 13.9 months. Specifically, it was 30.3 ± 14.2 months for CU participants and 31.1 ± 13.5 for those with MCI. There were no statistically significant differences observed in all plasma biomarkers in CU and MCI between the subgroups with and without cardiovascular disease, hypercholesterolemia, hypertension, and diabetes (*p* > 0.05; Figure S1 in Supplementary Materials). The only exception was GFAP, which was significantly increased in CU participants with hypercholesterolemia (*p* = 0.01) and a marginally significant decrease in MCI participants with diabetes (*p* = 0.5; Figure S1 in Supplementary Materials). The mean and standard deviation of baseline variables is displayed in Table 1.

**Table 1:** Baseline characteristics of the study cohort by cognitive stage. Table denotes mean ± standard deviation for continuous variables and frequency (percentage) for dichotomous variables

Variables	CU (*n* = 140)		MCI (*n* = 78)		Total sample	*p*
**Demographics**	**Mean ± SD**	*n*	**Mean ± SD**	*n*	*N*	
Age, years	65 ± 8	140	72 ± 7.7	78	218	< 0.001
Gender, female	89 (64%)	140	36 (46%)	78	218	0.018
Education, years	16 ± 4	140	14 ± 4	78	218	0.002
MMSE	28.6 ± 1.4	140	26 ± 2.4	78	218	< 0.001
APOE carriers	36 (28%)	136	38 (49%)	68	204	< 0.001
**Imaging**	**Mean ± SD**	*n*	**Mean ± SD**	*n*	*N*	
Hippocampal volume	7838 ± 788	75	6983 ± 996	71	146	< 0.001
Amyloid PET positivity	9 (25%)	36	39 (57%)	77	113	0.008
Amyloid Centiloid	14.5 ± 32.8	35	41.7 ± 42.8	68	103	0.002
Tau PET positivity	1 (3%)	36	24 (35%)	69	105	0.001
Tau Global SUVR	1.16 ± 0.14	23	1.32 ± 0.26	57	80	0.014
**Plasma Biomarkers**	**Mean ± SD**	*n*	**Mean ± SD**	*n*	*N*	
Aβ42/Aβ40	0.064 ± 0.013	140	0.059 ± 0.013	78	218	0.009
p-tau181	13.2 ± 7	140	19.9 ± 10	78	218	< 0.001
p-tau231	7.8 ± 4.1	140	12.2 ± 6.1	78	218	< 0.001
NfL	15.8 ± 7.9	140	20.9 ± 9.3	78	218	< 0.001
GFAP	113 ± 55	140	187 ± 113	78	218	< 0.001

### Linear mixed effect models

#### Univariate models

**Fig. 1:**
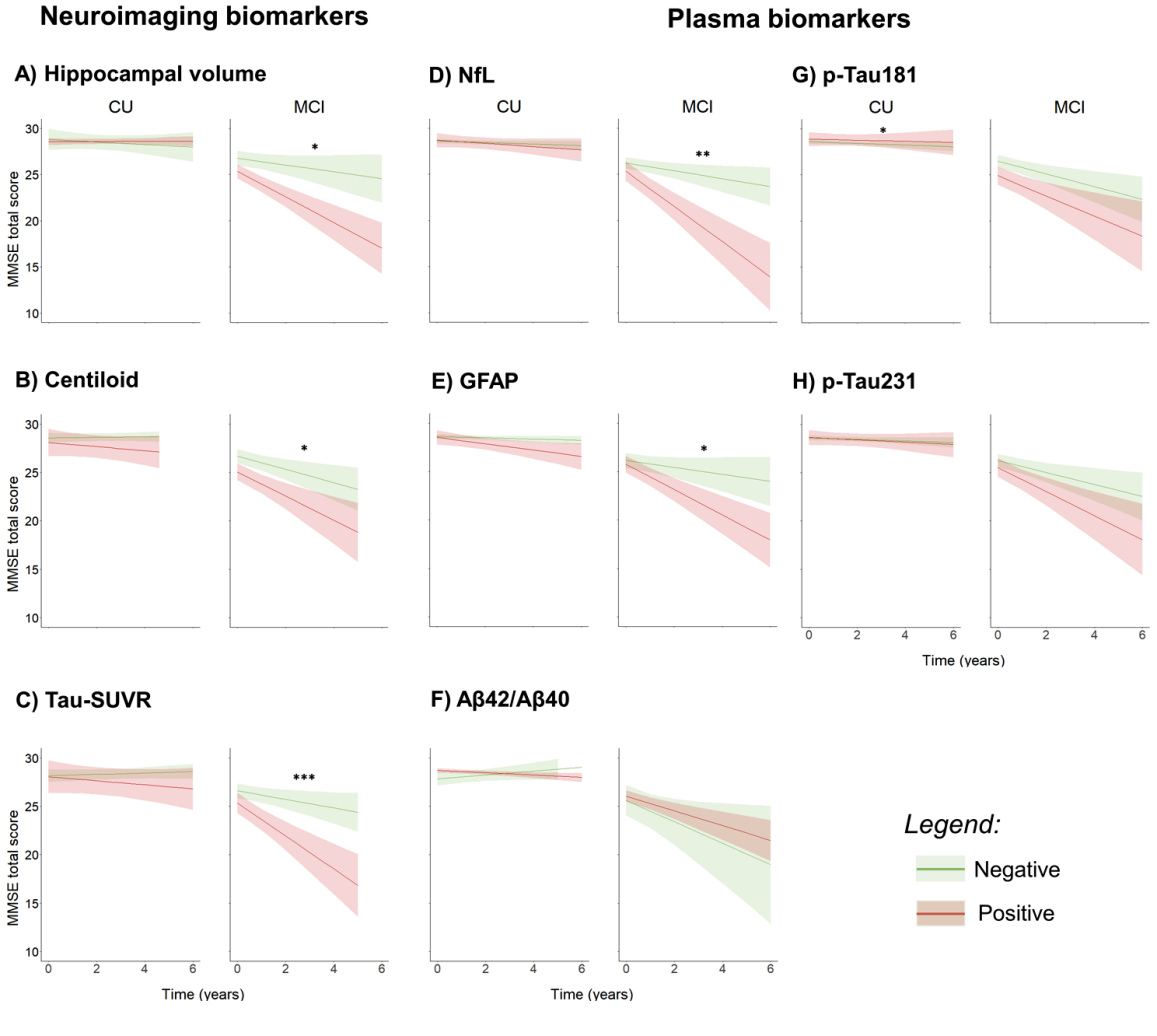
Linear mixed model prediction in both CU and MCI subset based on baseline neuroimaging and plasma biomarkers. Biomarker positivity in MCI and CU was defined based on the 90% percentile of the distribution in the CU. *** *p* < 0.001; ** *p* < 0.01; * *p* < 0.05

The univariate LME model in CU subjects revealed that only p-tau181 had significant interaction between the baseline level and time (β = 0.12, *p* = 0.02). The other biomarker models revealed no significant effect in the CU subjects (Table S2 in Supplementary Materials). In MCI, LME models revealed that cognitive decline was significantly predicted by NfL (β=-0.43, *p* = 0.006), GFAP (β=-0.38, *p* = 0.015), and a trend toward significance was observed in Aβ42/40 (β = 0.34, *p* = 0.051). Likewise, neuroimaging biomarkers significantly predicted cognitive decline, namely hippocampal volume (β = 0.44, *p* = 0.01), centiloid (β=-0.38, *p* = 0.04), and tau-SUVR (β=-0.66, *p* < 0.001). P-tau181 and p-tau231 values did not reveal a significant prediction of cognitive decline (Fig. 1; Table 2). Subgroup analysis in MCI patients positive to amyloid showed that GFAP (β=-1.32, *p* = 0.016) and NfL (β=-1.84, *p* < 0.001) significantly predicted cognitive decline, whilst for other biomarkers no significant interactions were found. All biomarkers revealed no significant results for MCI subjects negative to amyloid. In the Amy + CU group, the LME models also did not reveal any significant results in any biomarker.

**Table 2:** Linear mixed models of plasma and neuroimaging biomarkers predicting MMSE score change in MCI. The first columns represent the univariate model with each biomarker modelled individually, and the last columns represent the model comprising all the biomarkers. All the models included age, sex, and years of education as fixed factors

Predictors	Univariate model with one biomarker				Multivariate model with all biomarkers (AIC = 621)			Multivariate model with significant predictors (AIC = 715)		
	Estimate	Std. Error	*p*	AIC	Estimate	Std. Error	*p*	Estimate	Std. Error	*p*
**Plasma biomarkers**										
p-Tau181	-0.15	0.17	0.38	944	0.59	0.32	0.06			
p-Tau231	-0.19	0.29	0.28	957	-0.15	0.38	0.69			
Aβ42/Aβ40	0.34	0.18	0.05	1002	-0.12	0.22	0.59			
GFAP	-0.38	0.16	0.01	996	0.18	0.29	0.53			
NfL	-0.43	0.16	0.006	1000	-0.8	0.31	0.009	-0.42	0.17	0.02
**Neuroimaging biomarkers**										
Hippocampal volume	0.44	0.18	0.01	921	0.17	0.25	0.49			
Centiloid	-0.38	0.19	0.04	874	-0.06	0.3	0.84			
Tau-SUVR	-0.66	0.19	0.001	715	-0.71	0.31	0.02	-0.55	0.17	0.002

#### Multivariate models

The multivariate model in CU subjects revealed a marginally significant effect of p-tau181 (β = 0.29, *p* = 0.08). No other significant results were found in CU subjects. In MCI, the total LME model revealed that only NfL (β=-0.8, *p* = 0.009) and tau-SUVR (β=-0.71, *p* = 0.02) significantly predicted cognitive decline (Fig. 1; Table 2). Additionally, the multivariate LME comprising only the significant biomarkers in MCI, revealed similar results than univariate models, namely tau-SUVR (β=-0.55, *p* = 0.002) had a slightly larger coefficient than NfL (β=-0.42, *p* = 0.02). The model fit of the multivariate model comprising all the plasma and neuroimaging biomarkers (AIC = 621) was higher than all univariate models (AIC for each biomarker in Table 2), and also than the multivariate with significant predictors (AIC = 715).

### Cox proportional-hazards regression model

The categorization method labeled a total of 11 cognitive decliners, namely 10 of them in the MCI group and one amongst the CU. All biomarkers had non-significant effects in CU subjects, which can be explained by the low number of cognitive decliners.

**Fig. 2:**
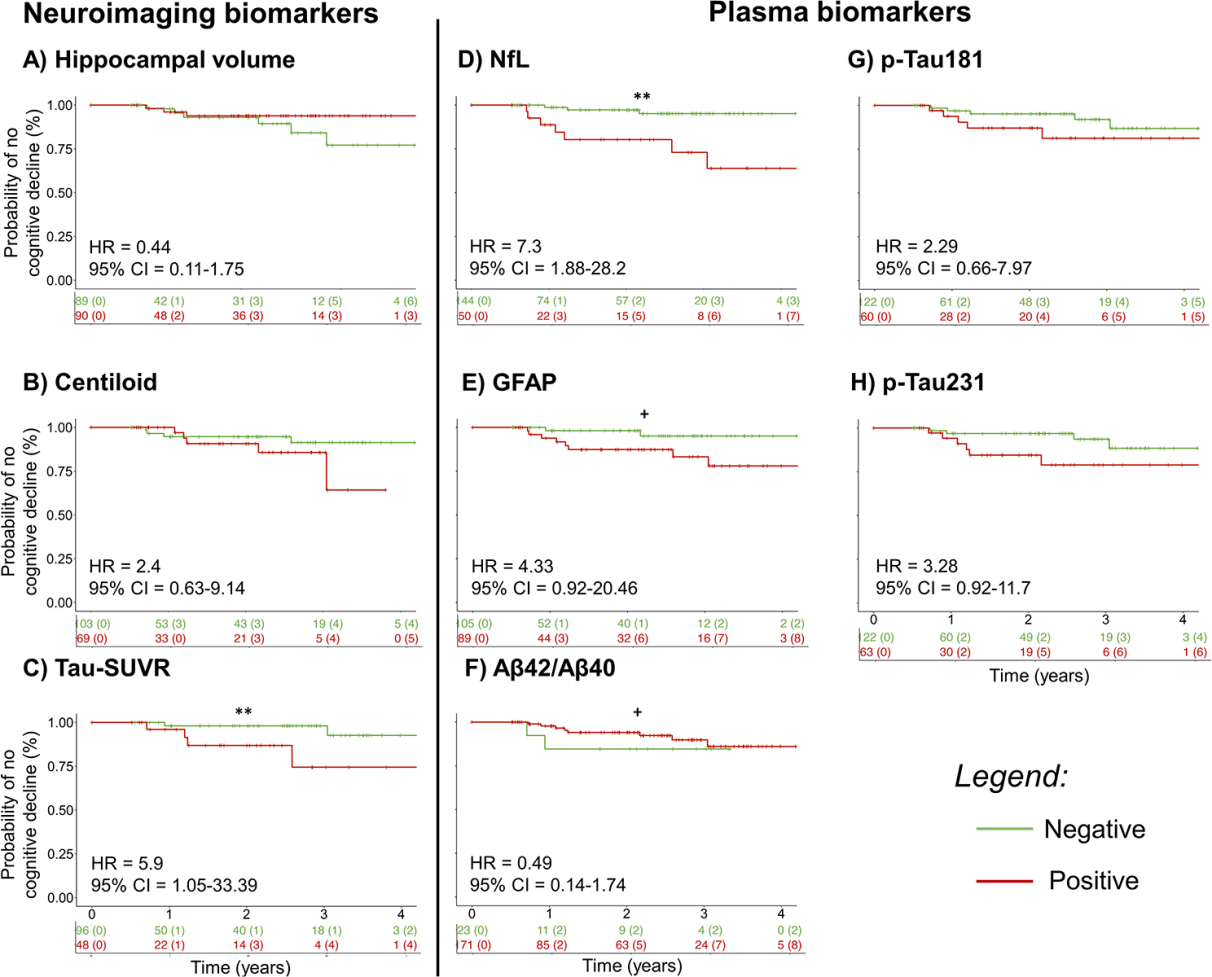
Kaplan-Meier Survival Curve plot showing the survival probability of cognitive decline in MCI over time considering the positivity in plasma and neuroimaging biomarkers. ** *p* < 0.01; * *p* < 0.05; + *p* < 0.1

CPH analysis revealed that baseline NfL (HR = 7.28, 95%CI = 1.88–28.2, *p* = 0.004) and tau-SUVR (HR = 5.9, 95%CI = 1.05–33.39, *p* = 0.04) were a significant predictor of converting to “cognitive decliner” in MCI. In line with this, baseline GFAP (HR = 4.33, 95%CI = 0.92–20.46, *p* = 0.06), and p-tau231 (HR = 3.28, 95%CI = 0.92–11.7, *p* = 0.07) had a trend toward significance. At last, no significant effect was detected in baseline p-tau181 (HR = 2.29, 95%CI = 0.66–7.97, *p* = 0.19), Aβ42/40 (HR = 0.49,95%CI = 0.14–1.74, *p* = 0.46), hippocampal volume (HR = 0.44, 95%CI = 0.11–1.75, *p* = 0.24), and centiloid (HR = 2.4, 95%CI = 0.63–9.14, *p* = 0.2) (Fig. 2).

### Sample size calculation for future AD clinical trials

A clinical trial with Amy + MCI participants aiming a reduction of 30% of cognitive decline will need 164 participants per arm. If we additionally consider GFAP+, the sample size will reduce two-fold and 84 participants per arm will be needed. On the other hand, if we consider Amy+/ NfL + subjects the sample size will be reduced by a factor of 4 because the estimation is 38 participants per arm. Lastly, if we consider both NfL+/ GFAP+, the results will be very similar to considering only NfL+. Moreover, the number of participants needed to screen varies among the biomarkers. For instance, a preventive trial studying a reduction of 30% of cognitive decline in MCI participants will need to screen 167 Amy + subjects per arm. If we add GFAP + to the inclusion criteria, we will need to screen 142 subjects per arm and 106 subjects per arm if we consider NfL+.

**Fig. 3:**
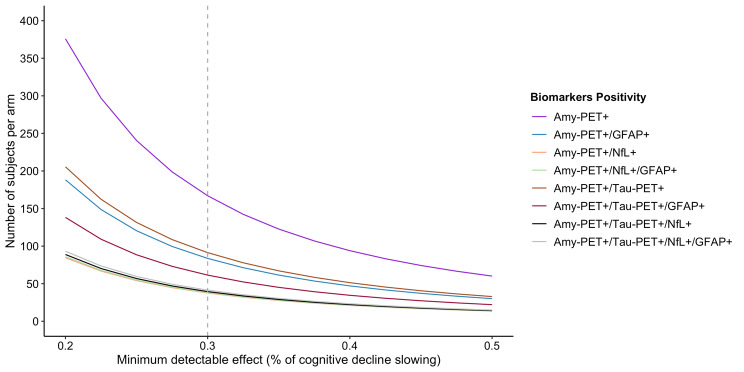
Number of amyloid positive MCI subjects per arm for preventive clinical trials with different percentages of cognitive decline slowing considering the positivity of plasma biomarkers in subjects positive to Amy-PET only or combined with Tau-PET

Considering a trial enrolling Amy+/Tau + participants to detect a 30% slowing of cognitive decline, the study will need 100 participants per arm. In the case of adding GFAP+, the sample size will decrease to 64 participants per arm, whereas the inclusion of NfL + will decrease the sample size to 39 participants per arm. Likewise, the number of subjects to screen will decrease, namely, the Amy+/Tau + participants needed to screen will be 204 per arm, whereas the inclusion of GFAP + in screening will decrease this number to 185 and NfL + to 139. All the results for other percentages of cognitive decline are reported in Table 3; Fig. 3.

**Table 3:** Number of participants to enroll and screen for future AD clinical trials. The sample size calculation was based on the LME models for subjects positive to amyloid-PET combined with tau-PET and/or plasma biomarkers positivity. The number of participants per arm was estimated for several percentages of cognitive decline slowing (measured as MMSE points/year) and the number of participants to screen were also calculated based on the positivity rate for each biomarker

Positivity based on	Linear mixed effect models				Number of participants per arm according to the % of cognitive decline slowing (number of participants to screen)			
	*N* out of 39 amy-positive MCI participants	Inter-subject variability of random slope	Residual variance	Random slope (cognitive decline)	20%	30%	40%	50%
Amy-PET	39 (100%)	0.68	2.02	-1.07	375 (375)	167 (167)	94 (94)	60 (60)
Amy-PET/NfL	14 (36%)	0.55	1.92	-2.14	85 (236)	38 (106)	21 (58)	14 (39)
Amy-PET/GFAP	23 (59%)	0.61	1.97	-1.47	188 (319)	84 (142)	47 (80)	30 (51)
Amy-PET/NfL/GFAP	12 (31%)	0.56	1.94	-2.13	86 (277)	38 (123)	22 (71)	14 (45)
Amy-PET/Tau-PET	19 (49%)	0.68	1.9	-1.36	226 (461)	100 (204)	56 (114)	36 (100)
Amy-PET/Tau-PET/NfL	11 (28%)	0.51	1.92	-2.05	89 (317)	39 (139)	22 (79)	14 (50)
Tau-PET/GFAP	13 (33%)	0.57	1.98	-1.69	138 (418)	61 (185)	35 (106)	22 (67)
Tau-PET/NfL/GFAP	10 (26%)	0.52	1.94	-2.02	93 (358)	41 (158)	23 (88)	15 (58)

## Discussion

This study suggests that plasma GFAP and NfL levels, and neuroimaging biomarkers (i.e., centiloid, tau-SUVR, hippocampal volume) can predict cognitive decline in MCI subjects. Specifically, the most accurate neuroimaging and plasma biomarkers for predicting cognitive deterioration were NfL and tau-SUVR. These results were also supported by the prognostic values suggesting a higher likelihood of cognitive decline in subjects positive to NfL and global tau-SUVR. Finally, we showed that evaluating NfL and GFAP levels in future AD clinical trials could significantly reduce the number of subjects to enroll.

In line with our results, NfL has been suggested as a strong predictor of cognitive changes among plasma biomarkers, such as Aβ42/40 [11, 13, 15] or total tau [31]. In fact, our results also suggest a higher predictive power for NfL when compared to p-tau181 and p-tau231. Previous literature demonstrated that increased NfL levels were associated with N measures (i.e., brain atrophy, hypometabolism) [32], which might explain a stronger relationship with the cognitive outcome according to the ATN model. Our findings also showed that GFAP was able to predict a decrease in global cognition, even though of a smaller extend than NfL. However, recent works found similar effects between both biomarkers [17], thus, the inferiority of GFAP comparing to NfL in the predictive effect of long-term cognitive deterioration is still not clear. This might be explained by the fact that plasma GFAP might represent an earlier marker than NfL in the AD continuum, and it has been suggested to mediate the relationship between amyloid and tau pathologies in preclinical AD subjects [33]. Nonetheless, this is of particular interest because both NfL and GFAP were shown to be increased also in non-AD neurodegenerative disorders [13, 34] such as frontotemporal dementia [35]. Thus, even though NfL and GFAP are biomarkers non-specific to AD, the cognitive decline predicted by both can be explained by their association with neurodegeneration and neuroinflammation, respectively [13, 36]. Likewise, our subgroup analysis in amyloid positive subjects also suggests that both biomarkers can be used to the prognosis of AD progression. Overall, both biomarkers may play a significant role in memory clinic samples since multiple etiologies may be contributing to cognitive deterioration.

In contrary to both previously mentioned biomarkers, plasma Aβ42/40 has failed to predict cognitive decline in CU and MCI subjects [13, 15–17]. Previous literature has suggested that plasma Aβ42/40 is mostly increasing during the preclinical AD stage [37], while NfL and GFAP levels increase along the symptomatic stages of AD [2]. Nonetheless, our findings reveal a trend towards significance (i.e., *p* < 0.1), thus a smaller predictive power of Aβ42/40 when compared to NfL and GFAP. For p-tau181 and p-tau231, we did not observe the expected predictive effect in cognition found in previous studies with CU [13] and MCI subjects [15]. Intriguingly, we found a small negative association between p-tau181 and cognitive decline in CU subjects. This finding highlights the fact that p-tau181 is a more sensitive biomarker in later disease stages [16]. It is important to point out that despite the presence of a statistically significant effect in CU, the differences detected between the first and the last MMSE evaluation were very small in magnitude (Fig. 1). A possible explanation is that the MMSE test may not be sensitive enough to detect subtle changes in the CU population, leading to increased variability (see Limitations section). Moreover, p-tau181 levels could be influenced by other health factors such as chronic kidney disease [24], which were not considered in this study but commonly observed in memory clinic populations (for other co-morbidities analysis see Figure S1 in Supplementary Materials). A recent longitudinal study has shown minimal changes of p-tau181 and p-tau231 in Aβ-positive CU and MCI individuals, despite large baseline changes [38] This change is better represented by p-tau217 which was associated with atrophy and cognitive measure over 8-years [38]. In fact, both p-tau181 and p-tau231 have demonstrated inferior diagnostic accuracy in identifying amyloid and tau in comparison with p-tau217 [39].

Our results are also in line with previous literature suggesting that tau-SUVR was found to be the strongest predictor among neuroimaging biomarkers of cognitive deterioration [29]. Moreover, previous studies also showed that tau-SUVR was the best predictor when compared to CSF biomarkers (i.e., Aβ42, p-tau181, t-tau) [9]. Although in our study neuroimaging biomarkers such as centiloid and hippocampal volume also revealed significant predictions of cognitive changes, their predictive effect was not as strong as NfL and tau-SUVR. The prognostic values estimated in CPH models also suggested NfL and tau-SUVR as the strongest predictors of cognitive decline. Given that measures of tau and neurodegeneration are substantially related with cognitive outcome, these findings are consistent with the ATN model [1].

Lastly, our findings are also important in future preventive AD clinical trials because they allow a proper identification of the target population [40]. We demonstrated that identifying NfL + in the group of Amy + subjects would result in a fourfold reduction in sample size, whereas if we identified GFAP + we could reduce it by twofold. The addition of NfL + to the inclusion criteria of trials enrolling subjects positive for amy-PET and tau-PET can also reduce the sample size by half. The decrease in sample size is a result of include NfL and GFAP in the inclusion criteria, as these markers are indicative of a higher probability of cognitive decline. Additionally, considering the significant reduction of subjects to enroll by the implementation of plasma biomarkers, we also demonstrated that is possible to reduce the number of subjects to screen. Our findings are in line with previous sample size calculations using other neurodegeneration measures for AD clinical trials [41]. Thus, the use of plasma biomarkers of neurodegeneration and neuroinflammation to identify Amy + subjects with a higher likelihood of declining cognitively should be considered a good research practice in future clinical trials, allowing a more efficient use of resources.

### Limitations

In terms of limitations, this study was not able to confirm the predictive effect in CU subjects already demonstrated in the literature [11–13, 42]. The lower sample size and the lack of long-term follow-ups might explain this result. Firstly, our CU cohort was composed of 140 participants, whereas previous studies had sample sizes ranging from 150 to 564 participants. Additionally, the maximum follow-up in our CU cohort was 5.7 years and the median was 2.2 years, only 53 subjects had follow-up later than 3-years. Lastly, MMSE might not be the most suitable neuropsychological test in CU participants given its low sensitivity in this population, which might benefit with other neuropsychological composites such as Preclinical Alzheimer Cognitive Composite [43]. Overall, the assessment of long-term effects in CU subjects has been highly influenced by these factors given that the decline in this population is not as steep as in the MCI stage.

Furthermore, the positivity in MCI subjects was estimated based on data from the CU subjects, considering the lack of approved cut-offs. As a result, the calculation of the statistical power analysis was influenced by the characteristics of our cohort. Therefore, future research should test for inter-individual differences in genotype or vascular factors in the prediction of cognitive decline. Nonetheless, our findings in sample size calculation were in line with the LME analysis, which did not consider biomarker positivity. Likewise, our comparison between plasma and neuroimaging biomarkers is limited by the fact that the population size is not identical for all analyses. We also acknowledge a vast array of p-tau immunoassays are described in the literature, with different properties, which might have influenced our result [44].

## Conclusion

This study suggests that plasma NfL and GFAP could be cost-effective prognostic biomarkers in the AD continuum. Although both biomarkers are not specific for AD pathology, they can assume an important role in the prediction of cognitive decline in amyloid-positive subjects. Specifically, NfL showed a similar result to tau-SUVR and was superior when compared to other plasma and neuroimaging biomarkers. Our findings were based on a unique memory clinic sample but suggesting their applicability to improve the diagnostic workup. In addition, the implementation of plasma biomarkers can help decreasing the samples sizes of AD clinical trials.

### Electronic supplementary material

Below is the link to the electronic supplementary material.

Supplementary Material 1

## Data Availability

No datasets were generated or analysed during the current study.

## Declarations

### Author access to data

All authors had full access to all the data in the study and takes responsibility for the integrity of the data and the accuracy of the data analysis.
