# Supplementary material for: Comparison of plasma and neuroimaging biomarkers to predict cognitive decline in non-demented memory clinic patients
Source: Alzheimers Res Ther. 2024 May 16;16:110. doi: 10.1186/s13195-024-01478-9 (PMC11097559; doi:10.1186/s13195-024-01478-9)
Supplement: Supplementary file 1 — Supplementary Material 1 [file 13195_2024_1478_MOESM1_ESM.docx]

**Comparison of plasma and neuroimaging biomarkers to predict cognitive decline in non-demented persons**

Augusto J. Mendes, PhD; Federica Ribaldi, PhD, Aurelien Lathuiliere, MD, PhD; Nicholas J. Ashton, PhD; Henrik Zetterberg, MD, PhD; Marc Abramowicz, MD; Max Scheffler, MD; Frédéric Assal, MD; Valentina Garibotto, MD; Kaj Blennow, MD; Giovanni B. Frisoni, MD

**CONTENT**

**Formula S1. Sample size calculation.**

**Figure S1**. Boxplots for each plasma biomarker in CU and MCI according to their medical history in cardiovascular diseases, hypercholesterolemia, hypertension, and diabetes (A-D) and scatter plot between each plasma biomarker and creatinine levels (E).

**Figure S2**. Individual lines of the trajectory of cognitive decline in MMSE scores over time based on baseline neuroimaging (A-C) and plasma biomarkers (D-H).

**Table S1. Linear mixed models of plasma and neuroimaging biomarkers predicting MMSE score change in MCI subjects considering the raw data.** The first columns represent the univariate model with each biomarker modelled individually, and the last columns represent the model comprising all the biomarkers. All the models included age and years of education as fixed factors.

**Table S2. Linear mixed models of plasma and neuroimaging biomarkers predicting MMSE score change in CU subjects.** The first columns represent the univariate model with each biomarker modelled individually, and the last columns represent the model comprising all the biomarkers. All the models included age, sex and years of education as fixed factors.

**Formula S1. Sample size calculation**

$\frac{n}{arm}= 2\left( z_{\left\{ 1-\frac{\alpha}{2} \right\}}+ z_{\left\{ 1-\beta\right\}} \right)^{2}\times\frac{\left( \sigma_{b}^{2}+\frac{\sigma_{e}^{2}}{\sum\left( t_{i}- \bar{t} \right)^{2}} \right)}{\left( \delta^{2}- \theta^{2} \right)}$

*z*_1−α/2_ and *z*_1−β/2_ represent the quantiles of the standard normal distribution of type I error rate (α) and statistical power (1-β). σ*_b_* and σ*_e_* denote, respectively, the inter-subject variability in random slopes and the residual error of variance from the LME models. The number of follow-ups and the mean time between them are *t*_i_ and $\bar{t}$*.* Lastly, the minimum detectable effect is represented by the δ, while the slope of the cognitive decline detected in the LME model is represented by ϴ. The values of each variable are reported in Table 3 of the manuscript.


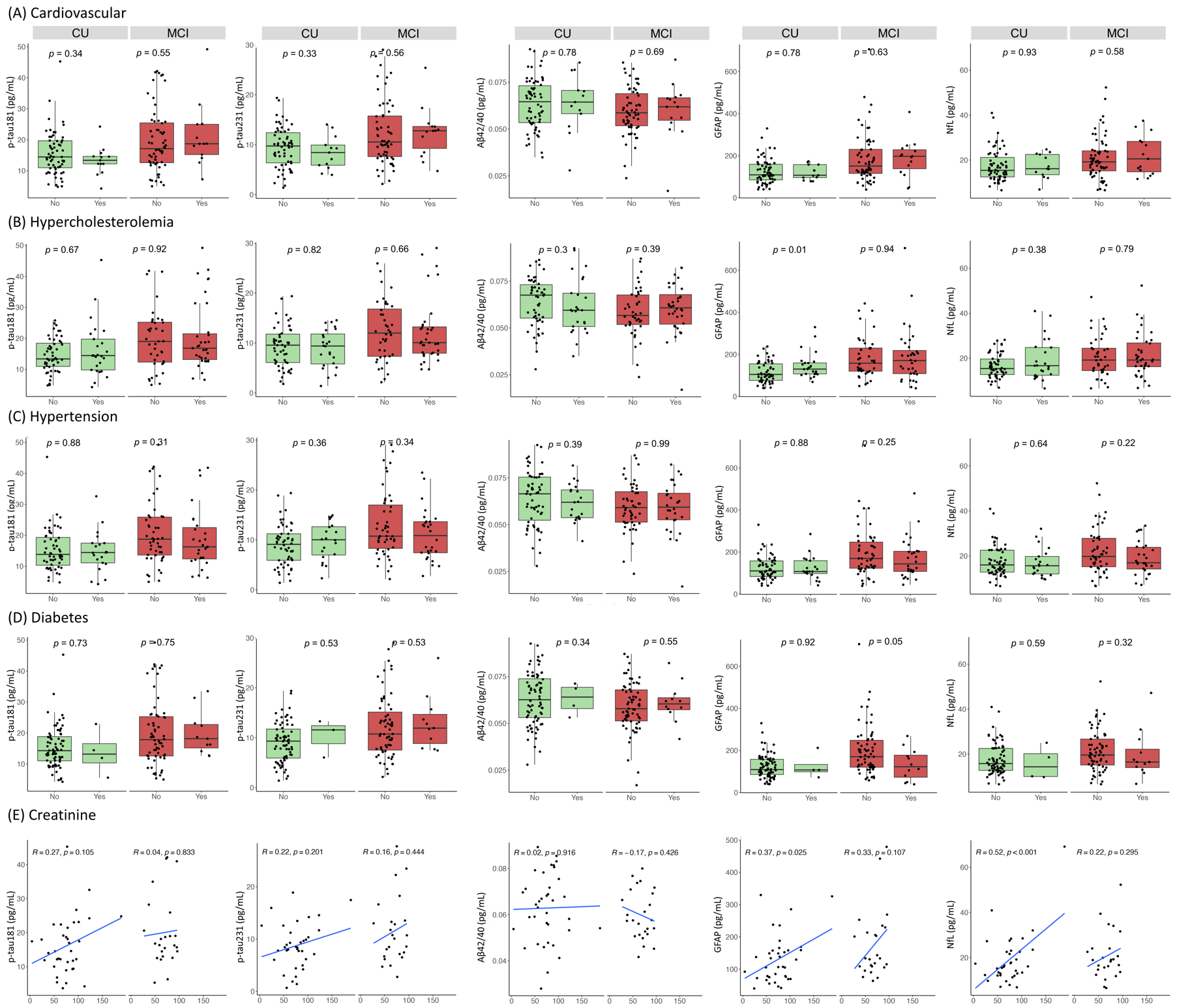


**Figure S1**. Boxplots for each plasma biomarker in CU and MCI according to their medical history in cardiovascular diseases, hypercholesterolemia, hypertension, and diabetes (A-D) and scatter plot between each plasma biomarker and creatinine levels (E).


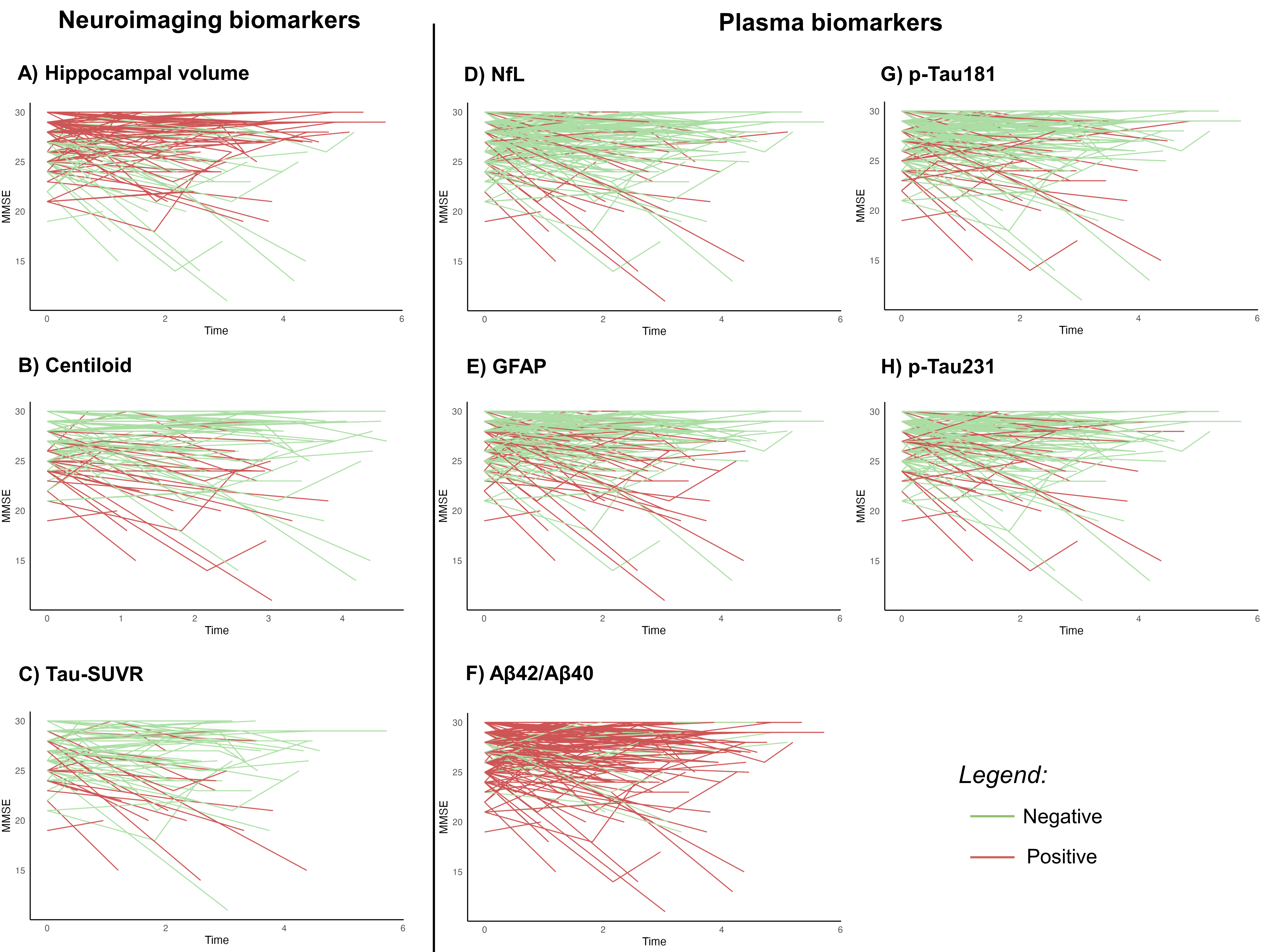


**Figure S2**. Individual lines of the trajectory of cognitive decline in MMSE scores over time based on baseline neuroimaging (A-C) and plasma biomarkers (D-H).

**Table S1. Linear mixed models of plasma and neuroimaging biomarkers predicting MMSE score change in MCI subjects considering the raw data.** The first columns represent the univariate model with each biomarker modelled individually, and the last columns represent the model comprising all the biomarkers. All the models included age, sex, and years of education as fixed factors.

|  |  |  |  |  |  |  |  |  |  |  |  |  |  |
| --- | --- | --- | --- | --- | --- | --- | --- | --- | --- | --- | --- | --- | --- |
| **Predictors** | **Univariate model with one biomarker (raw data)** | | | |  | **Multivariate model with all biomarkers (raw data scaled; AIC = 626)** | | |  | **Multivariate model with significant predictors (raw data scaled; AIC = 714)** | | |  |
|  | **Estimate** | **Std. Error** | ***p*** | **AIC** |  | **Estimate** | **Std. Error** | ***p*** |  | **Estimate** | **Std. Error** | ***p*** |  |
| **Plasma biomarkers** |  |  |  |  |  |  |  |  |  |  |  |  |  |
| p-Tau181 | -0.01 | 0.02 | 0.48 | 954 |  | 0.63 | 0.34 | 0.07 |  |  |  |  |  |
| p-Tau231 | -0.03 | 0.03 | 0.28 | 964 |  | -0.16 | 0.41 | 0.69 |  |  |  |  |  |
| Aβ42/Aβ40 | 29.74 | 12.55 | 0.02 | 981 |  | -0.1 | 0.19 | 0.61 |  |  |  |  |  |
| GFAP | -0.003 | 0.001 | 0.02 | 1018 |  | 0.04 | 0.18 | 0.83 |  |  |  |  |  |
| NfL | -0.06 | 0.02 | 0.001 | 1003 |  | -0.85 | 0.26 | 0.0009 |  | -0.45 | 0.16 | 0.007 |  |
| **Neuroimaging biomarkers** |  |  |  |  |  |  |  |  |  |  |  |  |  |
| Hippocampal volume | 0.0004 | 0.0001 | 0.01 | 950 |  | 0.09 | 0.24 | 0.71 |  |  |  |  |  |
| Centiloid | -0.008 | 0.004 | 0.04 | 889 |  | -0.11 | 0.28 | 0.69 |  |  |  |  |  |
| Tau-SUVR | -2.56 | 0.74 | 0.0005 | 709 |  | -0.58 | 0.29 | 0.04 |  | -0.54 | 0.18 | 0.003 |  |
|  |  |  |  |  |  |  |  |  |  |  |  |  |  |

**Table S2. Linear mixed models of plasma and neuroimaging biomarkers predicting MMSE score change in CU subjects.** The first columns represent the univariate model with each biomarker modelled individually, and the last columns represent the model comprising all the biomarkers. All the models included age, sex and years of education as fixed factors.

| **Predictors** | **Univariate model with one biomarker** | | | |  | **Multivariate model with all biomarkers (AIC = 186)** | | |
| --- | --- | --- | --- | --- | --- | --- | --- | --- |
|  | **Estimate** | **Std. Error** | ***p*** | **AIC** |  | **Estimate** | **Std. Error** | ***p*** |
| **Plasma biomarkers** |  |  |  |  |  |  |  |  |
| p-Tau181 | 0.12 | 0.05 | 0.02 | 989 |  | 0.29 | 0.16 | 0.08 |
| p-Tau231 | -0.01 | 0.05 | 0.8 | 965 |  | -0.17 | 0.37 | 0.64 |
| Aβ42/Aβ40 | -0.05 | 0.04 | 0.29 | 1025 |  | 0.12 | 0.17 | 0.46 |
| GFAP | -0.02 | 0.05 | 0.61 | 1027 |  | 0.05 | 0.2 | 0.8 |
| NfL | -0.03 | 0.04 | 0.57 | 1030 |  | -0.22 | 0.24 | 0.37 |
| **Neuroimaging biomarkers** |  |  |  |  |  |  |  |  |
| Hippocampal volume | 0.06 | 0.06 | 0.29 | 587 |  | 0.19 | 0.22 | 0.4 |
| Centiloid | -0.001 | 0.07 | 0.99 | 279 |  | 0.03 | 0.18 | 0.88 |
| Tau-SUVR | -0.1 | 0.08 | 0.24 | 198 |  | -0.1 | 0.17 | 0.54 |
|  |  |  |  |  |  |  |  |  |
